# Effect of EDTA Conditioning and Carbodiimide Pretreatment on the Bonding Performance of All-in-One Self-Etch Adhesives

**DOI:** 10.1155/2015/141890

**Published:** 2015-10-19

**Authors:** Shipra Singh, Rajni Nagpal, Shashi Prabha Tyagi, Naveen Manuja

**Affiliations:** ^1^Department of Conservative Dentistry and Endodontics, Kothiwal Dental College and Research Centre, Moradabad 244001, India; ^2^Department of Pediatric Dentistry, Kothiwal Dental College and Research Centre, Moradabad 244001, India

## Abstract

*Objective*. This study evaluated the effect of ethylenediaminetetraacetic acid (EDTA) conditioning and carbodiimide (EDC) pretreatment on the shear bond strength of two all-in-one self-etch adhesives to dentin. *Methods*. Flat coronal dentin surfaces were prepared on one hundred and sixty extracted human molars. Teeth were randomly divided into eight groups according to two different self-etch adhesives used [G-Bond and OptiBond-All-In-One] and four different surface pretreatments: (a) adhesive applied following manufacturer's instructions; (b) dentin conditioning with 24% EDTA gel prior to application of adhesive; (c) EDC pretreatment followed by application of adhesive; (d) application of EDC on EDTA conditioned dentin surface followed by application of adhesive. Composite restorations were placed in all the samples. Ten samples from each group were subjected to immediate and delayed (6-month storage in artificial saliva) shear bond strength evaluation. Data collected was subjected to statistical analysis using three-way ANOVA and post hoc Tukey's test at a significance level of *p* < 0.05.  *Results and Conclusion*. EDTA preconditioning as well as EDC pretreatment alone had no significant effect on the immediate and delayed bond strengths of either of the adhesives. However, EDC pretreatment on EDTA conditioned dentin surface resulted in preservation of resin-dentin bond strength of both adhesives with no significant fall over six months.

## 1. Introduction

Adhesion to dentin may be achieved either following an “etch-and-rinse” or a “self-etch” approach. Self-etch approach has been claimed to be user-friendlier and less technique-sensitive. Another important clinical benefit of self-etch adhesives is the absence of, or at least lower incidence of postoperative sensitivity [1]. This has been attributed to their less aggressive and more superficial interaction with dentin leaving tubules largely obstructed by smear [2]. However, some studies have shown a potential disadvantage in incorporating the smear layer into the hybrid layer [3–5]. Although the smear layer is reinforced by impregnated resin, bonding defects may be produced [6]. Since such defects may decrease the resistance and stability of the hybridized smear layer, its removal by incorporating a separate etching step may be necessary to obtain reliable, strong resin-dentin bonds [7–9]. Therefore, a conditioning system capable of changing the tooth surface by removing the smear layer and partially removing the surface layer of hydroxyapatite while simultaneously not destroying the organic portion of the dentin may be beneficial as pretreatment for mild self-etch adhesives. Some studies have demonstrated that separate phosphoric acid etching of dentin could decrease the bond strength and durability of self-etch adhesives [10, 11]. Therefore, conditioning with a mild etchant like EDTA may prove to be beneficial for bonding of mild self-etch adhesives to dentin.

Whereas phosphoric acid etching of dentin leads to dissolving both the extra and the intrafibrillar minerals resulting in recession and collapse of the collagen matrix, only partial removal of the smear layer with the maintenance of about 30% of the smear plugs and no morphological alterations of the dentin surface is observed following application of 17% EDTA on dentin for 60 seconds [12]. Phosphoric acid-etching of dentin causes collagen fibrils to become slightly denatured and swollen compared to EDTA-treatment [13].

Jacques and Hebling reported that pretreatment with a mild etchant such as 0.5 M EDTA improved the bond strength of the Clearfil SE bond [14]. Torii et al. also reported that EDTA conditioning was effective in improving dentin bonding for all-in-one adhesives [15]. Therefore, it may be anticipated that EDTA conditioning may improve the bonding efficacy of mild all-in-one self-etch adhesives to dentin [16]. Moreover EDTA has been shown to have a MMP inhibitory effect which may help in improving the durability of resin-dentin bond [17].

Degradation of the resin-dentin bonds, due to hydrolysis of the collagen fibrils, involves the participation of endogenous matrix metalloproteinases (MMPs) which become entrapped within the dentin substrate during tooth development [18, 19]. Therefore, the use of MMP inhibitors and collagen cross-linkers have been suggested as a valid alternative in an attempt to prolong the resin-dentin bond stability by overcoming this self-degradation process [20]. Chlorhexidine (CHX), Galardin (GL), CMT, SB-3 CT, proanthocyanidins (PA), 1-ethyl-3-(3-dimethylaminopropyl) carbodiimide (EDC), tetracycline, and quaternary ammonium methacrylates or benzalkonium chloride have been employed in various studies to improve the durability of resin-dentin bond [21–25].

The potential of cross-linkers is related to the possibility to improve the mechanical strength of the collagen network, improve the resistance to enzymatic degradation, and inactivate exposed MMPs bound to matrix collagen. When acid-etched dentin containing activated matrix-bound MMPs is treated with cross-linking agents, they inactivate the catalytic site of proteases [26]. Carbodiimide [EDC, 1-ethyl-3-(3-dimethylaminopropyl)] has been described as a collagen cross-linker with MMP inhibitory properties. EDC have been used as alternative cross-linking agents to glutaraldehyde, since they contain no potentially cytotoxic aldehyde residuals [27, 28]. EDC effectively improves the durability of resin-dentin bonds by increasing the mechanical properties of the collagen matrix [29]. Most of the research regarding the effect of EDC on dental adhesion has been done using etch-and-rinse adhesives; however the effect of EDC on bonding of contemporary self-etch adhesives needs to be evaluated. Moreover the effect of prior EDTA conditioning on the bonding of specific all-in-one adhesive systems still needs to be determined. Hence, the aim of this study was to investigate the effect of (i) EDTA conditioning, (ii) EDC pretreatment, or (iii) combined effect of EDTA preconditioning and EDC application on the immediate and long-term bonding efficacy of two different all-in-one self-etch adhesives. The null hypothesis tested was that there is no effect of EDTA or EDC pretreatment on the immediate and delayed bond strength of two different all-in-one self-etch adhesives to dentin.

## 2. Materials and Method

The study was performed in one hundred and sixty freshly extracted noncarious, human molars. The teeth were examined under stereomicroscope (Olympus, Tokyo, Japan) and teeth free of caries, cracks, or any developmental defects were included. Teeth were cleaned of debris. Calculus was removed using ultrasonic scaler and then the teeth were stored in 0.5% Chloramine T Trihydrate (Sigma Aldrich, Bangalore, India) for no more than 3 months. Tooth crowns were flattened using a low-speed diamond saw (Isomet, Buehler Ltd., Lake Bluff, IL, USA) under water irrigation unless superficial dentin was visible and a standardized smear layer was created with 600-grit silicon-carbide (SiC) paper. The samples were embedded in an autopolymerizing resin at the level of cementoenamel junction with long axis perpendicular to the acrylic resin surface. Teeth were randomly divided into eight groups according to two different self-etch adhesives used (G-Bond (GC Corp., Tokyo, Japan) and OptiBond-All-In-One (KERR, Orange, CA, USA)) (Table 2) and four different surface pretreatments. Each group was further divided into two subgroups for immediate (a) and delayed (b) bond strength evaluation.

*Group 1* (GB). G-Bond was applied following manufacturer's instructions.

*Group 2* (GB-EDTA). Dentin conditioning with 24% EDTA gel for 1 minute (Trisodium EDTA Gel, Pyrex Pharmaceuticals, Roorkee), followed by rinsing with distilled water, blot dried prior to application of G-bond.

*Group 3* (GB-EDC). Application of EDC (0.3 M for 1 minute) on smear covered dentin surface and blot dried before application of G-Bond.

*Group 4* (GB-EDTA + EDC). Dentin was conditioned with 24% EDTA gel for 1 minute, rinsed with distilled water, and blot dried. This was followed by application of EDC (0.3 M) for 1 minute and then blot dried, followed by application G-Bond adhesive.

*Group 5* (OB). OptiBond-All-In-One was applied following manufacturer's instructions.

*Group 6* (OB-EDTA). Dentin conditioning with EDTA (24% gel for 1 minute) followed by rinsing with water blot dried prior to application of OptiBond-All-In-One.

*Group 7* (OB-EDC). Application of EDC (0.3 M for 1 minute) on smear covered dentin surface and blot dried before application of OptiBond-All-In-One.

*Group 8* (OB-EDTA + EDC). Dentin was conditioned with 24% EDTA gel for 1 minute, rinsed with distilled water, and blot dried. This was followed by application of EDC (0.3 M) for 1 minute and then blot dried, followed by application OptiBond-All-In-One adhesive.

Transparent plastic tubes 54-HL (TYGON Medical tubing, Saint Gobain, Akron, OH, USA) of internal diameter 3 mm and 2 mm height with thickness 0.5 mm were precut and placed perpendicular to the prepared surface. A hybrid resin composite (Filtek Z350 XT, Body Shade A1, Nanohybrid, 3 M ESPE) was filled into the precut tubes. Each bonded specimen was light-cured for 20 seconds using Spectrum 800 (Dentsply, Caulk, Milford, USA) at light intensity of 600 mW/cm^2^. The plastic tubes were gently cut and carefully removed with a number 11 surgical blade after polymerization.

## 3. Determination of Dentin Shear Bond Strength

Half of the specimens (1a–8a) were then stored in distilled water at 37°C for 24 hours for immediate testing. The remaining half samples from each group (1b–8b) were stored in artificial saliva (ICPA, Wet Mouth) for 6 months before shear bond strength evaluation [30, 31]. Shear bond strength was determined using a universal testing machine (Instron, ADMET, Enkay Enterprises, New Delhi) using the corresponding computer software. The specimens were placed and stabilized by the jig, while a straight knife-edge rod (2.0 mm) was applied at the tooth restoration interface at a crosshead speed of 1 mm/minute. Load was applied until restoration failure. The mode of failure was determined by observation under a stereomicroscope (Olympus, Tokyo, Japan) at 10x magnification and classified into adhesive (A), mixed (M), and cohesive (C) failures in either dentin or resin. The statistical analysis was done using three-way ANOVA and post hoc Tukey's test SPSS 16.0 version (Statistical Package, SPSS Inc., Chicago, IL, USA) at a significance level of *p* < 0.05.

## 4. Results

**Figure 1:**
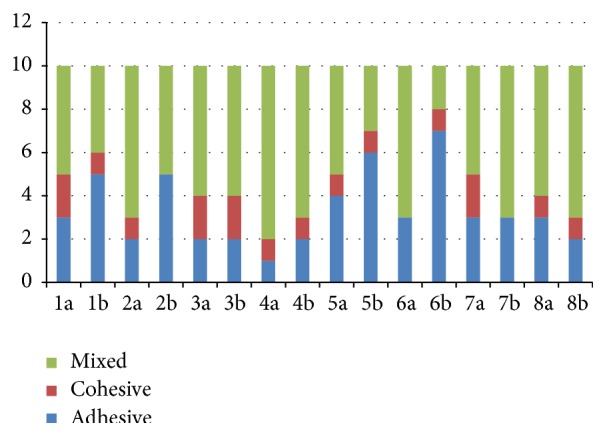
Failure modes in different experimental groups.

Mean shear bond strength values and standard deviation of all the groups are presented in Table 1. There was no significant difference in bond strengths between the two adhesives when used according to manufacturer's instructions (Table 2). EDTA pre-conditioning had no significant effect on the immediate bond strength of either of the adhesives (Groups 1a and 2a; *p* = 0.707; Groups 5a and 6a; *p* = 0.959). EDC pretreatment alone also had no significant effect on the immediate bond strength of G-Bond (Groups 1a and 3a; *p* = 0.836) and OptiBond-All-In-One (Groups 5a and 7a; *p* = 0.999). Mixed fractures were the most common failure mode in all the groups (Figure 1). There was no significant difference in the mode of failure between the two tested adhesives.

There was a significant reduction in bond strength for both adhesives G-Bond and OptiBond All-In-One after six months storage (Groups 1a and 1b; *p* = 0.022; Groups 5a and 5b; *p* = 0.020). EDTA preconditioning could not prevent the fall in bond strength over a six-month storage period (Groups 2a and 2b; *p* = 0.018; Groups 6a and 6b; *p* = 0.006). However, EDC pretreatment alone (Groups 3a and 3b; *p* = 0.429; Groups 7a and 7b; *p* = 0.344) or EDC application on EDTA conditioned dentin surface (Groups 4a and 4b; *p* = 0.286; Groups 8a and 8b; *p* = 0.403) resulted in preservation of resin-dentin bond strength of both the adhesives with no significant fall over six months. Failure mode analysis revealed mixed fractures to be the most common in Groups 3b : 4b; 7b and 8b. An increase in adhesive fractures was observed in Groups 1b : 2b; 5b : 6b (Figure 1).

## 5. Discussion

In the present study, no significant difference was observed between the immediate shear bond strength values of G-Bond and OptiBond-All-In-One adhesives and mixed fractures were the most common failure mode. Bond strength of polymerized adhesives depends upon various factors such as the type of cross-linking monomers, presence and type of filler particles, degree of conversion, and the amount of residual organic solvents. The carboxylic group of 4-MET (4-methacryloyloxyethyl trimellitic acid) renders G-Bond monomers hydrophilic, but less reactive than UDMA (urethane dimethacrylate) in hydrogen bonding with water and it functions as a proton donor that bonds ionically with calcium in hydroxyapatite crystalites [32, 33]. Thus, an extremely thin interface nanointeraction zone (300 nm) is formed as opposed to the traditional hybrid layer appellation that provides resistance to acute debonding stresses and better bond durability and survival of adhesion, minimizing voids. Strong air blowing of the primed surface as suggested in G-Bond accelerates the evaporation of solvent-acetone and the resultant water droplets formed due to phase separation. The excess of nonpolymerizable hydrophilic components (water, acetone, and glutaraldehyde) may give rise to hydration forces that repel water at film boundaries and hence less water sorption [32]. Aromatic rings present in G-Bond are more stable [34].

OptiBond-All-In-One contains 35 to 45% acetone and 4–9% ethanol. The solvent evaporation from adhesives is influenced by the vapor pressure [35]. As the vapor pressure of acetone is high, it volatilizes rapidly and may dehydrate the dentin. The presence of water in self-etch adhesives is necessary to ensure the ionization of the acidic monomers, but it is not as efficient as acetone or ethanol as a solvent because of its lower vapor pressure [36]. The presence of acetone and ethanol in OptiBond-All-In-One might balance the solvent evaporation without dehydrating dentin, because ethanol ensures the wetness of the substrate, and its vapor pressure is intermediate between acetone and water. Another explanation for the good performance of OptiBond-All-In-One could be the content of glycerol phosphate dimethacrylate monomer in its formulation, a surfactant monomer that may have facilitated the penetration of hydrophobic components into dentin, reducing the phase separation [37, 38]. G-Bond is HEMA (2-hydroxyethyl methacrylate) free adhesive whereas OptiBond-All-In-One is HEMA containing adhesive [33, 39–41]. The hydrophilic monomer, HEMA, in various concentrations is frequently added to one-step self-etch adhesives because of its positive influence on adhesion to dentin, the miscibility of hydrophilic and hydrophobic components in the adhesive blend, and prevention of phase separation. The hydrophilic monomer of HEMA tends to cluster together before polymerization, leading to creation of hydrophilic domains. Moreover, HEMA attracts water even after polymerization. When HEMA is cured in the presence of water, polymerization is incomplete and a porous hydrogel is formed that allows water to permeate through the adhesive layer, compromising bonding effectiveness. It was reported that the amount of water sorption of adhesive polymers increased proportionally to their HEMA concentrations [39]. Some studies have shown that the removal of HEMA from self-etch adhesives would minimize water sorption, while others have observed that the 10% HEMA content would be beneficial for the adhesive system performance [39]. There is still a controversy about the role of HEMA in the bonding performance of adhesives. In the study by Felizardo et al. [39] it was concluded that the influence of HEMA on bond strength to dentin was material dependent.

As there are numerous factors involved in bond degradation, several methods have been proposed (i.e., load cycling, thermal cycling, prolonged water, and artificial saliva incubation) for reproducing clinical situations and simulating the oral environment to test the durability of dentin bonding [42]. In our study, after storage in artificial saliva for six months, both G-Bond and OptiBond-All-In-One depicted significant reduction in the bond strength when used without any pretreatment. Several studies have provided morphological evidence of resin elution and/or hydrolytic degradation of collagen matrices after long-term storage [43]. Accordingly, more adhesive failures were observed after 6-month period.

EDTA is a molecule containing four carboxylic acid groups that can chelate calcium. It has been widely used to dissolve the mineral phase of dentine without altering dentin proteins, thereby avoiding major alterations of the native fibrillar structure of dentin collagen. Further, EDTA has an inhibitory effect on the matrix-bound MMPs of demineralized dentin [17]. In the current study, EDTA preconditioning had no significant effect on the immediate bond strength of the tested self-etch adhesives. Kasraei et al. reported that EDTA application before one-step self-etch adhesive significantly improved the bond strength [44]. However, they used liquid EDTA at 0.5 M concentration for 30 seconds and the adhesives evaluated were also different from our study. Soares et al. also depicted increased bond strength of self-etch adhesive systems used with EDTA preconditioning [45]. However, they conducted the study on bovine incisors using two-step self-etch adhesives whereas, in our study, one-step all-in-one adhesives were used. It has been reported that the efficiency of EDTA depends on many factors: penetration depth of the material, hardness of the dentin, duration of application, pH, form (liquid or gel), and concentration of material [42]. Although EDTA is an excellent MMP inhibitor, it is also water soluble; hence it might be rinsed off EDTA-treated dentin [17]. This might not be able to sustain MMP inhibition for much longer duration. Therefore, in the current study no improvement in durability could be observed after EDTA pretreatment with significant reduction in bond strength after six months, along with an increase in the number of adhesive failures. Another important aspect that must be considered is EDTA delivery form. Even at a higher concentration, a 24% EDTA gel might not be able to etch dentin in the same manner as EDTA in aqueous solution due to its lower wetting capacity. Stape et al. evaluated the effect of 24% EDTA on bond strength of resin cements to dentin and concluded that the effect varied with the different resin cements [46]. Parihar and Pilania also concluded that the effect of EDTA preconditioning on bonding of self-adhesive resin cement was product dependent [47].

EDC, a cross-linking agent with very low cytotoxicity, has shown promising results in eliminating dentin collagen degradation and preserving dentin bond strength with clinically acceptable procedure time [48]. It is the most stable cyanamide isomer, which is able to assemble amino acids into peptides. They are examples of zero length cross-linking agents. However, application of EDC alone in this study, on the dentin surface, without prior EDTA conditioning had no significant effect on the immediate bond strength of both self-etch adhesives. Probably as there was no exposed collagen, EDC was not able to strengthen the collagen matrix by increased cross-linking, whereas most of the studies, which report improved bonding effectiveness with EDC, have been performed using etch and rinse adhesives where EDC is applied to dentin previously demineralized by phosphoric acid which exposes the collagen fibrils.

EDTA removes the smear layer and mildly demineralizes the dentin. Because EDTA does not denature collagen in comparison to phosphoric acid, it creates thinner hybrid layers that are more easily infiltrated with resin [49–52]. Conditioning with 24% EDTA for 1 minute has been shown to demineralize the dentin and expose the collagen fibrils. Subsequent application of EDC promotes cross-linking amongst exposed collagen fibrils. Thus, in the present study, dentin pretreatment with EDC (with and without EDTA) resulted in bond strength preservation after 6 months of storage in artificial saliva for both adhesives used. All EDC treated groups at 6 months revealed mixed fracture patterns to be the most common failure mode. A previous in vitro study also reported that EDC application for 1 minute was effective in inactivating soluble rhMMP-9 and matrix-bound dentin proteinases [53].

Our results are supported by the study of Mazzoni et al., who reported preservation of resin-dentin bond with 1 minute EDC pretreatment and by the study of Bedran-Russo et al. who also reported increased durability of resin-dentin bonds in EDC pretreated group [54, 55]. EDC is capable of cross-linking proteins through covalent peptide bonds by activating the free carboxyl group of glutamic and aspartic acids present in protein molecules to form O-acylisourea intermediate that reacts with the epsilon amino group of lysine or hydroxylysine in an adjacent polypeptide chain to form a stable amide cross-link [56, 57]. Cross-linking increases the mechanical properties of dentin collagen and makes the fibrils more resistant to degradation [55]. Furthermore EDC shows no transdentinal cytotoxicity on odontoblast-like cells and is able to increase the mechanical properties of the collagen matrix [25, 58]. Further studies are required to evaluate the effect of different concentration, time, pH, and form of application of EDTA and EDC on the long-term bonding efficacy of different contemporary adhesive systems to dentin. Effect of incorporation of EDC in the adhesive composition on the resin polymerization also needs to be investigated.

## 6. Conclusion

Carbodiimide pretreatment of dentin surface resulted in significant preservation of resin-dentin bond over six-month storage period for both all-in-one self-etch adhesives tested. EDTA pretreatment of dentin surface before application of self-etch adhesives had no effect on the durability of resin-dentin bond.

## Figures and Tables

**Table 1:** Mean shear bond strength values both immediate and delayed for G-Bond and OptiBond-All-In-One adhesives.

Immediate Testing			Delayed Testing			*p* value
Groups	Mean	SD	Groups	Mean	SD	
1a (GB)	33.30^abc^	5.54	1b	23.10^c^	5.53	0.022^*∗*^
2a (GB-EDTA)	38.40^ab^	9.23	2b	27.90^ab^	8.97	0.018^*∗*^
3a (GB-EDC)	37.70^abc^	7.72	3b	32.70^ab^	8.11	0.429
4a (GB-EDTA + EDC)	40.80^a^	7.58	4b	34.90^a^	5.49	0.286
5a (OB)	29.00^c^	6.99	5b	20.30^c^	5.83	0.020^*∗*^
6a (OB-EDTA)	32.30^abc^	4.79	6b	22.30^c^	7.45	0.006^*∗*^
7a (OB-EDC)	30.20^bc^	5.29	7b	25.60^bc^	5.52	0.344
8a (OB-EDTA + EDC)	31.90^abc^	6.44	8b	27.60^abc^	6.96	0.403

Groups with the same superscripts are not statistically different (*p* > 0.05);  *∗* denotes statistically significant groups.

**Table 2:** Composition and manufacturer's instructions of adhesive systems used in the study.

Adhesive	Composition	Manufacture	Technique
G-Bond	4-MET, phosphate ester monomer, UDMA, acetone, water, microfiller, and photoinitiator	GC Corp.; Tokyo, Japan	(i) Shake the bottle thoroughly prior to dispensing (ii) Immediately apply to the prepared enamel and dentin surfaces using the disposing applicator (iii) Leave undisturbed for 5–10 seconds (iv) Dry thoroughly for 5 seconds with oil free air under maximum air pressure. The final results should be a thin, rough, adhesive film with the appearance of frosted glass and which doesn't visibly move under further air pressure (v) Light cure for 10 seconds

OptiBond-All-In-One	GPDM, GDM, HEMA, Bis-GMA, water, ethanol, acetone, silica, CQ, and sodium hexafluorosilicate	OP; Kerr; Orange, CA, USA	(i) Shake adhesive bottle briefly. (vigorously for 10 seconds) (ii) Using the disposable applicator brush, apply a generous amount of OptiBond-All-In-One adhesive to enamel/dentin surface. Scrub the surface with a brushing motion for 20 seconds (iii) Apply a second application of OptiBond-All-In-One All-In-One adhesive with a brushing motion for 20 seconds (iv) Dry the adhesive with gentle air first and then medium air for at least 5 seconds with oil-free air (v) Light cure for 10 seconds
